# A Comparative Observational Study of the Use of Saline Uterine Hydrosonography for the Diagnosis and Assessment of Uterine Cavity Lesions in Women

**DOI:** 10.1155/2016/9317194

**Published:** 2016-08-11

**Authors:** Veluppillai Vathanan, Nii Adjeidu Armar

**Affiliations:** ^1^Frimley Health NHS, Wexham Park Hospital, Wexham SL2 4HL, UK; ^2^Central Middlesex Hospitals, Acton Lane, Park Royal, London NW10 7NS, UK

## Abstract

Aim of this study was to evaluate the performance of saline hydrosonography (HSGM) (also known as saline infusion sonography (SIS)) against transvaginal ultrasound scan (TVS) and hysteroscopy in the diagnosis of uterine cavity lesions. Diagnostic hysteroscopy with biopsy is considered as the “gold standard” to diagnose intrauterine abnormalities. The introduction of HSGM has improved the diagnostic capability of ultrasound. It is important to establish the efficacy and safety of HSGM before it is widely recommended for use. This retrospective observational data was collected from all 223 patients who underwent TVS, HSGM, and hysteroscopy as part of their gynaecological investigations from 1 January 2008 to 31 December 2010 at Central Middlesex Hospital, London.* Endometrial Polyps*. TVS: sensitivity 60.53%, specificity 97.06%, positive predictive value (PPV) 95.83%, and negative predictive value (NPV) 68.75% and HSGM: sensitivity 95%, specificity 97.14%, PPV 97.44%, and NPV 94.44%.* Submucous Leiomyoma*. TVS: sensitivity 57.14%, specificity 93.48%, PPV 84.21%, and NPV 78.18% and HSGM: sensitivity 96.55%, specificity 100.00%, PPV 100.00%, and NPV 97.92%. Diagnostic efficacy of HSGM is superior to TVS for the diagnosis of endometrial polyps and submucous fibroids. HSGM should be considered as an intermediate investigation after TVS to assess intracavity pathology and to confirm the diagnosis; hysteroscopy should become a therapeutic intervention.

## 1. Introduction

 Abnormal uterine bleeding accounts for approximately 20% of visits to gynaecology clinics and almost 25% of gynaecological operations in the UK [1].

40% of premenopausal women with abnormal uterine bleeding were found to have some intrauterine pathology [2]. Uterine fibroids (approximately 30%) and polyps (approximately 10%) are the most common form of pathology found [3].

A combination of diagnostic hysteroscopy with the histological examination of any endometrial aspirate or biopsy is considered as the “gold standard” for the diagnosis of intrauterine abnormalities [4].

Advances in ultrasound scanning technology have revolutionised the noninvasive diagnosis of uterine pathology. However, despite these developments, it is still not possible to visualize some problems with confidence, such as intrauterine adhesions [5]. Differentiating submucous fibroids from endometrial polyps can be difficult sometimes [6, 7]. Another example is distinguishing submucous fibroids which distort the cavity from those not distorting the cavity [8]. These differentiations become extremely important when the decision to treat and how to treat has to be made.

Uterine hydrosonography (HSGM) (also known as saline infusion sonography (SIS)) involves real time ultrasound visualization of the uterus during distension of the cavity with a sonolucent material which is usually sterile normal saline. The introduction of this technique has improved the diagnostic capability of ultrasound so that it has become a competitive method for visualization of uterine cavity pathology [9].

When compared to outpatient hysteroscopy, HSGM is less invasive [10], causes less pain and discomfort [11], carries no risk of uterine corporeal perforation, and is cheaper [12].

In spite of these advantages, HSGM is not widely available, neither is it much in use compared to TVS and hysteroscopy. We therefore considered it important to establish its efficacy and safety firmly before recommending its widespread use.

We have conducted this retrospective study to assess the diagnostic efficacy of HSGM as a tool for evaluating uterine intracavity lesions.

## 2. Materials and Methods

This is a retrospective observational study conducted at the Central Middlesex Hospital, London, which is a district general hospital serving a multiethnic population of North West London. The data was collected for a three-year period, from 1 January 2008 to 31 December 2010.

All the women who underwent a transvaginal ultrasound scan (TVS) evaluation together with a HSGM as part of their gynaecological investigations were identified from hospital data. Those who were suspected of having uterine intracavity lesions (by TVS or HSGM) underwent hysteroscopy and excision/biopsy of any suitable identified lesions.

The patient demographics, TVS, HSGM, hysteroscopy, and histology findings (if performed) were collected from the hospital patient records and from the hospital electronic reporting systems. Those with incomplete data were excluded from the analysis.

All TVS were performed by qualified sonographers in the ultrasound scan department. HSGM was largely performed by one consultant gynaecologist with a special interest in subfertility and a few were performed by senior trainees in gynaecology using a Sonoace 8000 SE ultrasound machine with a curvilinear transvaginal probe of frequency range 4 to 9 MHz.

A 5.2 FGr Goldstein catheter (Cook® Medical Inc, Ireland) was introduced into the uterine cavity and sterile 0.9% saline was injected via the catheter into the uterine cavity as the distension medium. All diagnostic hysteroscopies were performed under general anaesthesia using a 5 mm rigid, single flow, 30-degree-angle scope (Karl Storz, Tuttlingen, Germany) with sterile 0.9% saline as the distension medium.

Microsoft Excel 2010 and MedCalc version 11.5.1.0 were used for validation tests (sensitivity, specificity, and positive and negative predictive values) and analysis. Only those women who underwent all three procedures (TVS, HSGM, and hysteroscopy) were included in the validation test analysis.

## 3. Results

Out of 223 patients whose records were examined, 13 were excluded due to incomplete data; 210 patients were included in this analysis. Patient characteristics and the indication for the investigations are detailed in Table 1. 76 patients underwent hysteroscopy due to suspected intracavity lesions on HSGM and uterine cavity lesions were confirmed in 74 patients (Table 2).

There were no documented complications during or after the HSGM procedure in this study. The HSGM procedure was completed successfully in 99.52% of patients (1 HSGM was abandoned due to patient discomfort; however, a second attempt on a different day was successful).

TVS was “normal” in 32 patients. However, 9 of these were found to have intracavity pathology on HSGM (cervical polyp: 1, endometrial polyp: 4, submucous fibroid: 2, and Asherman's syndrome: 2).

Two women were diagnosed with intrauterine adhesions (Asherman's syndrome) by HSGM although the TVS failed to identify this problem in both cases.

Two women who were investigated for intermenstrual bleeding were found to have anterior isthmic defects (a consequence of previous lower segment caesarean section (LSCS)) by HSGM as the cause for their intermenstrual bleeding. Both of these isthmic defects were not identified by TVS.

The diagnostic efficacy of TVS and HSGM for the diagnosis of endometrial polyps and submucous fibroids were calculated by the validation tests shown in Table 3.

*For the Diagnosis of Endometrial Polyps*. TVS showed a sensitivity of 60.53%, specificity of 97.06%, and positive predictive value (PPV) and negative predictive value (NPV) of 95.83% and 68.75%, respectively. HSGM showed a sensitivity of 95%, specificity of 97.14%, PPV 97.44%, and NPV 94.44%.

*For the Diagnosis of Submucous Leiomyoma*. TVS showed a sensitivity of 57.14%, specificity 93.48%, PPV 84.21%, and NPV 78.18%, whereas HSGM showed a sensitivity of 96.55%, specificity 100.00%, PPV 100.00%, and NPV 97.92%.

## 4. Discussion

Our study confirms that HSGM has a higher sensitivity and specificity as well as positive and negative predictive values for the diagnosis of endometrial polyps and submucous fibroids when compared with TVS. It also confirms that the HSGM procedure has a low failure rate, in contrast to at least one other published study which claimed a failure rate of 7% [2].

Our lower failure rate could be due to the inclusion of very few postmenopausal women in our study (postmenopausal women, 1.5%) together with the high percentage of multiparous women in our study (63.34%). Studies have shown that postmenopausal status and nulliparity were associated with higher failure rates [4, 13, 14].

Another relevant factor may be operator experience as more than 98% of the HSGM procedures were performed by a single, experienced operator with more than 15 years' experience of performing HSGM. If this assumption is true, we suggest that the failure rate will fall further with increasing operator experience.

A systematic review of diagnostic hysteroscopy in abnormal uterine bleeding [15] concluded that the sensitivity and specificity of hysteroscopy to diagnose endometrial polyps was 0.94 (95% CI 0.92 to 0.96) and 0.92 (95% CI 0.91 to 0.94), respectively. For the diagnosis of submucous myomata, the sensitivity was 0.87 (95% CI 0.81 to 0.92) with a specificity of 0.95 (95%CI 0.93 to 0.97). The efficacy of hysteroscopy in their study is comparable to our findings for HSGM and it can therefore be concluded that HSGM is as effective as hysteroscopy for the diagnosis of endometrial polyps and submucous fibroids. This has been confirmed in other studies as well [16].

HSGM has the advantage of being less invasive and less expensive and it causes less patient discomfort and inconvenience. It also enabled us to quantify the lesions by measuring them and determining the position of the lesions accurately, relative to the myometrium [9]. During the uterine cavity examination, HSGM also provides an opportunity for the examiner to evaluate other uterine and pelvic structures at the same sitting.

The accuracy of the measurements obtained by the use of the sonolucent medium within the uterine cavity facilitates the choice of evidence-based treatment options. For example, the length of the uterine septum of up to 1 cm appears not to worsen reproductive performance and may not need surgical correction to improve reproductive outcome [17]. This can be accurately measured during HSGM but not quite as easily or readily using TVS alone.

HSGM identified 2 isthmic defects as the cause for intermenstrual bleeding (both missed by TVS). This is an important diagnosis for the management of these patients appropriately and, in these cases, the diagnosis was only secured by HSGM.

Patients tolerated the HSGM procedure well. Only 1 procedure out of 210 had to be abandoned due to patient discomfort. It also caused less inconvenience to the patients when compared to the impact of hysteroscopy as they were able to carry on with their normal daily routine immediately after the procedure.

This study confirmed that the diagnostic accuracy of HSGM for the diagnosis of endometrial polyps and submucous fibroids is as effective as hysteroscopy. Even though hysteroscopy offers the advantage of “see and treat” option, hysteroscopy (as both an outpatient and inpatient procedure) costs more than HSGM [11] and is more invasive.

Why should we choose an invasive option which causes more discomfort and involves more complications as well as a greater cost when the same objective can be achieved by a less invasive option? HSGM should therefore be considered as an intermediate procedure, before embarking on the more invasive hysteroscopy option. We are confident that the application of the HSGM technique in the management of abnormal uterine bleeding will eventually result in a significant reduction in the use of hysteroscopy (with its associated complications and greater costs) for diagnostic purposes.

### 4.1. Strengths and Limitations

One of the limitations was the retrospective collection of data. However, even though it was a retrospective study, the quality of data was extremely good. Out of 223 cases only 13 were excluded due to incomplete data. The findings are more representative for premenopausal than postmenopausal women as 98.5% of data was collected from premenopausal women.

However its value in postmenopausal women was established in a recent study [18] which concluded that HSGM is an easy to perform, safe, and well-tolerated procedure yielding high diagnostic accuracy in postmenopausal women.

## 5. Conclusion

It is clear from this study that the diagnostic efficacy of HSGM is superior to TVS for the diagnosis of endometrial polyps and submucous fibroids. When our findings are compared with that of the meta-analysis for hysteroscopy [18] it is obvious that HSGM is a competitive alternative to diagnostic hysteroscopy.

Given that HSGM is less invasive, carries no perforation risk, causes less discomfort and inconvenience, and costs less to carry out, it should be considered as an intermediate investigation after TVS to assess intracavity pathology and to confirm the diagnosis; hysteroscopy should become a therapeutic intervention and should primarily be reserved for this purpose in the future. A prospective observational study with cost evaluation is desirable to confirm the cost effectiveness of this approach.

## Figures and Tables

**Table 1:** Patient characteristics and indication for investigations.

Age in years, mean ± SD (range)	38.79 ± 7.68 (21–66)
	(Postmenopausal – 3 (1.5%))
Parity, median (range)	1 (0–8)
BMI, mean ± SD (range)	27.98 ± 5.99 (18–50)
Ethnicity, *n* (%)	
Asian	52 (27.46%)
Black Afro-Caribbean	95 (45.24%)
Caucasians	29 (13.81%)
Not recorded	22 (10.47%)
Others	12 (5.71%)

Principle presenting symptom, *n* (%)	
Menorrhagia	119 (56.667%)
Subfertility	23 (10.952%)
Chronic pelvic pain	17 (8.1%)
Intermenstrual bleed	12 (5.714%)
Dysmenorrhoea	10 (4.762%)
Irregular bleeding	14 (6.66%)
Other	15 (7.143%)

**Table 2:** Intracavity lesions confirmed by hysteroscopy.

Intracavity lesion	Numbers
Asherman's syndrome	2
Endometrial polyp	38
Endo. polyp + submucous fibroid	2
IUCD	2
Polypoid endometrium	3
Submucous fibroid	26

*Total*	*74*

**Table 3:** Validation tests for TVS and HSGM for the diagnosis of endometrial polyps and submucous fibroids.

			TVUSS		HSGM
			95% CI		95% CI
Endometrial polyps	Sensitivity	60.53%	43.39% to 75.96%	95.00%	83.08% to 99.39%
	Specificity	97.06%	84.67% to 99.93%	97.14%	85.08% to 99.93%
	Positive PV	95.83%	78.88% to 99.89%	97.44%	86.52% to 99.94%
	Negative PV	68.75%	53.75% to 81.34%	94.44%	81.34% to 99.32%

Submucous fibroids	Sensitivity	57.14%	37.18% to 75.54%	96.55%	82.24% to 99.91%
	Specificity	93.48%	82.10% to 98.63%	100.00%	92.45% to 100.00%
	Positive PV	84.21%	60.42% to 96.62%	100.00%	87.66% to 100.00%
	Negative PV	78.18%	64.99% to 88.19%	97.92%	88.93% to 99.95%

## References

[1] Goldstein S. R. (2004). Menorrhagia and abnormal bleeding before the menopause. *Best Practice & Research: Clinical Obstetrics and Gynaecology*.

[2] Emanuel M. H., Verdel M. J. C., Stas H., Wamsteker K., Lammes F. B. (1995). An audit of true prevalence of intra-uterine pathology: the hysteroscopical findings controlled for patient selection in 1202 patients with abnormal uterine bleeding. *Gynaecological Endoscopy*.

[3] NICE—National Institute for Clinical Excellence https://www.nice.org.uk/guidance/CG44.

[4] De Kroon C. D., De Bock G. H., Dieben S. W. M., Jansen F. W. (2003). Saline contrast hysterosonography in abnormal uterine bleeding: a systematic review and meta-analysis. *BJOG: An International Journal of Obstetrics and Gynaecology*.

[5] Allahbadia G. N., Kadam K., Allahbadia S. (2004). Saline infusion sonohysterography (SIS). *Reviews in Gynaecological Practice*.

[6] Clark T. J. (2004). Outpatient hysteroscopy and ultrasonography in the management of endometrial disease. *Current Opinion in Obstetrics & Gynecology*.

[7] Lalchandani S., Phillips K. (2004). Do we really need to hysteroscope all the women who have irregular bleeding on hormone replacement therapy?. *Gynecological Surgery*.

[8] Nanini R., Chelo R., Branconi F., Tantini C. (1994). Dynamic echohysterography: a new diagnostic technique. *ActaEurFertil*.

[9] Haimov-Kochman R., Pshitizky M., Hamani Y., Hurwitz A., Voss E. (2010). Hysterohydrosonoscopy—an integrated modality for uterine imaging. *Gynecological Surgery*.

[10] Van Dongen H., De Kroon C. D., Van den Tillaart S. A. H. M., Louwé L. A., Trimbos-Kemper G. C. M., Jansen F. W. (2008). A randomised comparison of vaginoscopic office hysteroscopy and saline infusion sonography: a patient compliance study. *BJOG: An International Journal of Obstetrics and Gynaecology*.

[11] Van Den Bosch T., Verguts J., Daemen A. (2008). Pain experienced during transvaginal ultrasound, saline contrast sonohysterography, hysteroscopy and office sampling: A Comparative Study. *Ultrasound in Obstetrics and Gynecology*.

[12] NICE Heavy menstrual bleeding costing report. https://www.nice.org.uk/guidance/cg44/resources/costing-report-195029821.

[13] Rogerson L., Bates J., Weston M., Duffy S. (2002). A comparison of outpatient hysteroscopy with saline infusion hysterosonography. *BJOG: An International Journal of Obstetrics and Gynaecology*.

[14] De Kroon C. D., Jansen F. W. (2006). Saline infusion sonography in women with abnormal uterine bleeding: an update of recent findings. *Current Opinion in Obstetrics and Gynecology*.

[15] van Dongen H., de Kroon C. D., Jacobi C. E., Trimbos J. B., Jansen F. W. (2007). Diagnostic hysteroscopy in abnormal uterine bleeding: a systematic review and meta-analysis. *BJOG: An International Journal of Obstetrics and Gynaecology*.

[16] Soguktas S., Cogendez E., Kayatas S. E., Asoglu M. R., Selcuk S., Ertekin A. (2012). Comparison of saline infusion sonohysterography and hysteroscopy in diagnosis of premenopausal women with abnormal uterine bleeding. *European Journal of Obstetrics Gynecology and Reproductive Biology*.

[17] Fedele L., Bianchi S., Marchini M., Mezzopane R., Di Nola G., Tozzi L. (1996). Residual uterine septum of less than 1 cm after hysteroscopic metroplasty does not impair reproductive outcome. *Human Reproduction*.

[18] Bingol B., Gunenc M. Z., Gedikbasi A., Guner H., Tasdemir S., Tiras B. (2011). Comparison of diagnostic accuracy of saline infusion sonohysterography, transvaginal sonography and hysteroscopy in postmenopausal bleeding. *Archives of Gynecology and Obstetrics*.

